# Penetration and scattering—Two optical phenomena to consider when applying proximal remote sensing technologies to object classifications

**DOI:** 10.1371/journal.pone.0204579

**Published:** 2018-10-09

**Authors:** Christian Nansen

**Affiliations:** Department of Entomology and Nematology, Davis, California, United States of America; Institute of Materials Science, GERMANY

## Abstract

Proximal remote sensing is being used across a very wide range of research fields and by scientists, who are often without deep theoretical knowledge optical physics; the author of this article falls squarely in that category! This article highlights two optical phenomena, which may greatly influence the quality and robustness of proximal remote sensing: penetration and scattering. Penetration implies that acquired reflectance signals are associated with both physical and chemical properties of target objects from both the surface and internal tissues/structures. Scattering implies that reflectance signals acquired from one point or object are influenced by scattered radiometric energy from neighboring points or objects. Based on a series of laboratory experiments, penetration and scattering were discussed in the context of “robustness” (repeatability) of hyperspectral reflectance data. High robustness implies that it is possible to control imaging conditions and therefore: 1) obtain very similar radiometric signals from inert objects (objects that do not change) over time, and 2) be able to consistently distinguish objects that are otherwise highly similar in appearance (size, shape, and color) and in terms of biochemical composition. It was demonstrated that robustness of hyperspectral reflectance data (40 spectral bands from 385 to 1024 nm) were significantly influenced by penetration and scattering of radiometric energy. In addition, it was demonstrated that the influence of penetration and scattering varied across the examined spectrum. Characterization of how optical phenomena may affect the robustness of reflectance data is important when using proximal remote sensing technologies as tools used to classify engineering and biological objects.

## Introduction

Proximal remote sensing has been defined as acquisition and classification of reflectance or transmittance signals with an imaging sensor mounted within a short distance (under 1 m and typically much less) from target objects [1]. When deploying proximal remote sensing, remittance (reflectance or transmission) signals are acquired under controlled light and temperature conditions and typically with a constant distance and angle between the imaging lens and target objects. The combination of: 1) high spectral resolution, 2) high spatial resolution, 3) high robustness of radiometric signals, 4) signal acquisition is non-invasive, and 5) processing of reflectance signals potentially being almost real-time—enables use and integration of proximal remote sensing into wide ranges of industrial operations with high throughput and also in many types of basic and applied engineering and biological research. Several reviews have described applications of proximal remote sensing in different research disciplines, including: food safety and food quality [2–6], biology of insects [1, 7], plant phenotyping and stress detection [8, 9], and pharmaceutical product analyses [10]. Separately, it is very important to highlight that the medical field has been studying and using proximal remote sensing technologies for over three decades [11]. Applications of proximal remote sensing in biomedical research was recently reviewed [12]. The basic purpose of proximal remote sensing is to demonstrate that remittance signals in selected spectral bands can be used to classify objects (food products, seeds, growing plants, insects, pills, etc) non-invasively. These applications of proximal remote sensing hinge on the assumption that unique and detectable remittance signals can be associated with specific qualitative traits or characteristics, so that the objects can be classified accurately and consistently. With the growing interest in applications of both proximal and airborne remote sensing technologies, it seems both timely and important to investigate some of the factors affecting the robustness of remittance signals.

“Robustness” [13] (also referred to as “spectral repeatability” [14]) of remittance signals is here defined as the level of consistency of acquired reflectance signals over time and space, and high robustness implies that, it is possible to control imaging conditions and therefore; 1) obtain very similar radiometric signals from inert objects (objects that do not change) over time, and 2) be able to consistently distinguish objects that are otherwise highly similar in appearance (size, shape, and color) and in terms of biochemical composition. The rapidly growing number of studies describing applications of proximal remote sensing is largely driven by the technology becoming progressively more robust, cost-effective, and also user-friendly. The latter means that scientists who come from a wide range of academic backgrounds become involved in applied proximal remote sensing applications without necessarily having the theoretical knowledge to appreciate the complexity and importance of phenomena associated with optical physics; the author of this article falls squarely in that category! We non-optical physicists see the value and potential of the technology through the lens of: 1) practical applications we encounter, 2) demand-driven research we propose in grant applications, 3) attempting to integrate novel technologies into university teaching, and 4) involving our students in meaningful and competitive capacity building for their career paths. This article was written with the intention to illustrate practical implications of two optical phenomena, which can have considerable influence on the quality and robustness of reflectance signals acquired with proximal remote sensing technologies: penetration and scattering of radiometric energy.

Penetration implies that, even though many applications of proximal remote sensing technologies refer to object surface reflectance, the acquired reflectance signal is associated with physical and chemical properties of the both the surface and underlying tissues or structure of target objects. In the medical field, the optical properties radiometric energy (light) have been well characterized for over three decades [11]. Moreover, the ability of radiometric energy to penetrate into soft human tissues (i.e. brain, liver, lung, skin) is used to characterize the function or structure of the tissues as part of disease diagnosis and image-guided surgery [12]. Scattering of radiometric energy from target objects means that the reflectance signal acquired from any given point is partially influenced by scattered radiometric energy from neighboring points or objects. The implications of scattering are that the reflectance signals acquired from a given object becomes relative to its proximity to other objects. The intensity and radiometric composition of scattering depends on the radiometric energy source and chemical and physical properties of the objects being imaged [15]. Scattering is also referred to as lens flare or veiling glare [16].

In this study only hyperspectral reflectance data will be considered (not transmission), and penetration and scattering were studied through a series of laboratory experiments with sheets of paper, plant leaves, candy [Skittles], and mosquito adults and eggs. Statistical comparisons were used to characterize the robustness of average reflectance values in individual spectral bands (40 spectral bands from 385 to 1024 nm). That is, no statistical effect of either penetration or scattering was considered indication of high robustness of reflectance data in a given spectral band. This series of experiments represents the first study, in which effects of both penetration and scattering were quantified across a wide radiometric range. These two optical phenomena were discussed in the context of robustness reflectance data and the reliability of proximal remote sensing technologies as tools used to classify engineering and biological objects.

## Materials and methods

### Hyperspectral imaging

Regarding all experimental data sets described below, hyperspectral reflectance data were acquired under environmental conditions similar to those described in previous studies [17–20]. In brief, a push-broom hyperspectral camera (PIKA XC, Resonon Inc., Bozeman, MT, USA) was mounted 20 cm above the specimens, and hyperspectral images were acquired with the spatial resolution of about 36 pixels per mm^2^ under artificial lighting (four 15W 12 V light bulbs with two on either side of the lens). The main specifications of the hyperspectral camera were: interface, Firewire (IEEE 1394b), digital output (14 bit), and angular field of view of 7 degrees. The objective lens had a 17 mm focal length (maximum aperture of F1.4), optimized for the near-infrared and visible near-infrared spectra. Originally, we acquired reflectance signals in 240 spectral bands from 385–1024 nm (spectral resolution = 2.1 nm), but these data were spectrally binned (averaged across six spectral bands) into 40 spectral bands (spectral resolution = 12.6 nm). During hyperspectral image acquisition, RH was between 30–40% and temperature 19–22°C in the lab. A piece of white Teflon (K-Mac Plastics, MI, USA) was used for white calibration. Reflectance value was referred to proportional reflectance and compared to reflectance obtained from white Teflon. All data processing and subsequent statistical analyses were conducted in PC-SAS 9.4 (SAS Institute, NC).

### Imaging of white paper placed on top of colored dots

**Fig 1:**
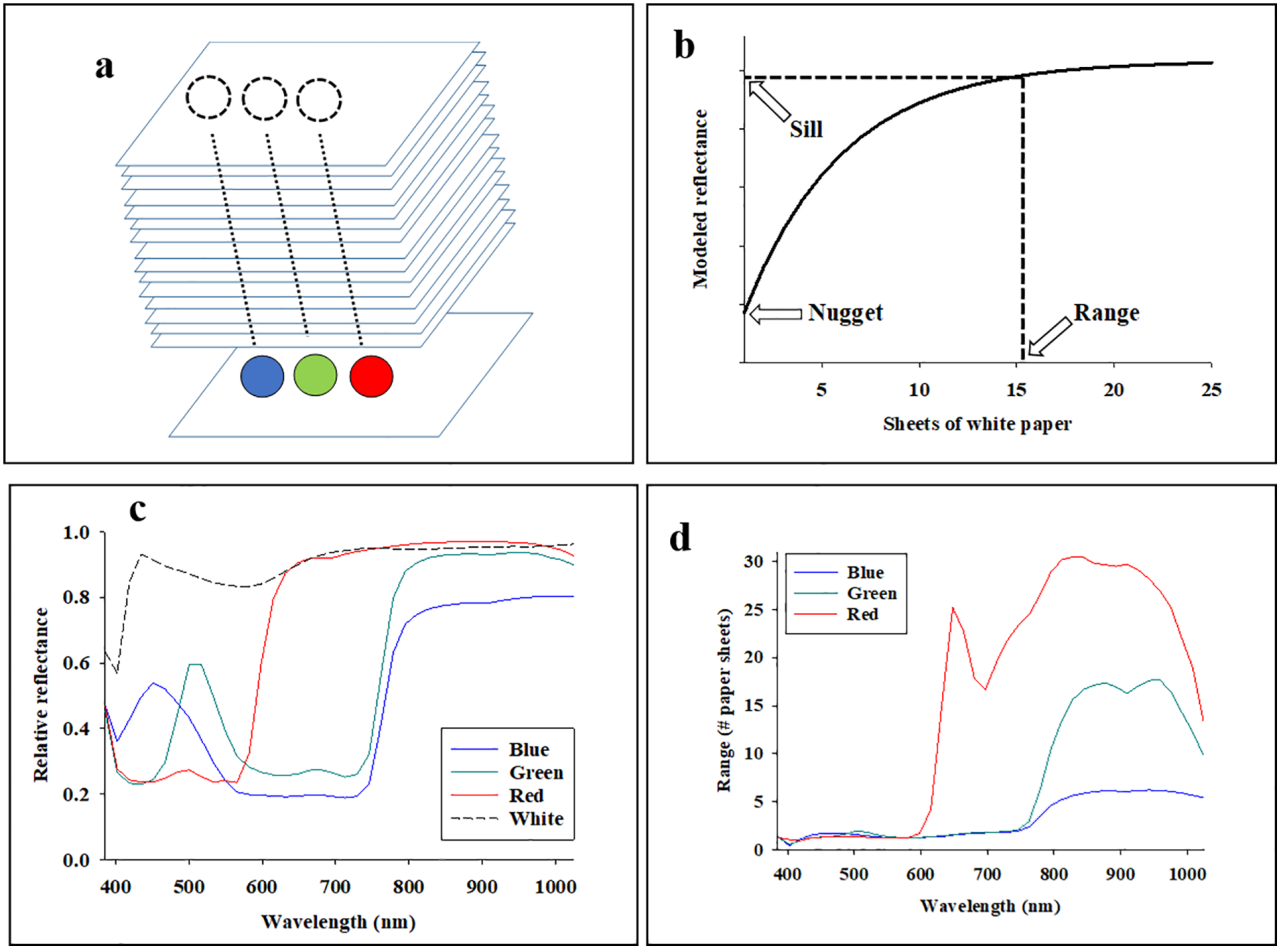
Experimental design with colored dots (blue, green, and red) on a white piece of paper were covered with 1–15 sheets of white paper (**a**). In Fig 1b-d, each cell represents the result from a statistical comparison of the average reflectance acquired immediately above the white dot and one of the colored dots [blue (**b**), green (**c**), and red (**d**)]. Color coding of cells indicates statistical difference (P < 0.05). In other words, a given wavelength was found to be sensitive to a specific background color (dot color) after sheets of white paper was placed on top of the colored dots.

To characterize penetration through layers of white paper sheets, standard grade office paper was used. A stack of 500 paper sheets equals 54 mm, so the average thickness per sheet was 0.108 mm. Average reflectance profiles were acquired from regions immediately above colored dots (blue, green, and red) (Fig 1a) after placing: 0, 1, 2, 3, 4, 5, 6, 8, 10, 12, 15, 20, and 25 additional sheets of white paper on top of the paper with colored dots. To ensure uniform packing of sheets of white paper, consistent weight was carefully placed on top of sheets of white paper around the region with colored dots. It was assumed that, as sheets of white paper were stacked on top of the colored dots, reflectance data acquired from sheets above colored dots would gradually stabilize (the effect of the colored dot would cease to affect the acquired reflectance data). In other words, and as depicted in Fig 1b, it was predicted that reflectance values in each spectral band would reach an asymptote after a certain number of white paper sheets had been placed on top of the colored dots. The asymptotic response of average reflectance in a specific spectral band (*R*_*x*_) was predicted as a function of number of white paper sheets (“*paper*”):

$$R_{x}=a+b\times [1-e^{\frac{-3\times paper}{c}}]$$ (1)

In Eq 1 above, there are three fitted coefficients, *a*, *b*, and *c*. This particular curve fit is widely used in spatial statistics and was selected, because the three fitted coefficients conveniently describe important characteristics of the asymptotic response [21]: 1) *a*: the intercept with the y-axis is denoted the “nugget”, 1) *b*: the asymptote is denoted the “sill”, and 3) *c*: the number of sheets of white paper when the sill is reached is denoted the “range”. Moreover, the range is here a direct estimate of how many sheets of white paper needed to eliminate the effect of the colored dot underneath. Using non-linear regression (proc nlin), Eq 1 was fitted to reflectance data from 0–25 sheets of white paper above the three colored dots (12 replicates), and separate non-linear regression analyses were conducted for all 40 spectral bands (three colored dots × 40 spectral band = 120 non-linear regression analyses).

### Imaging of magnolia leaves

**Fig 2:**
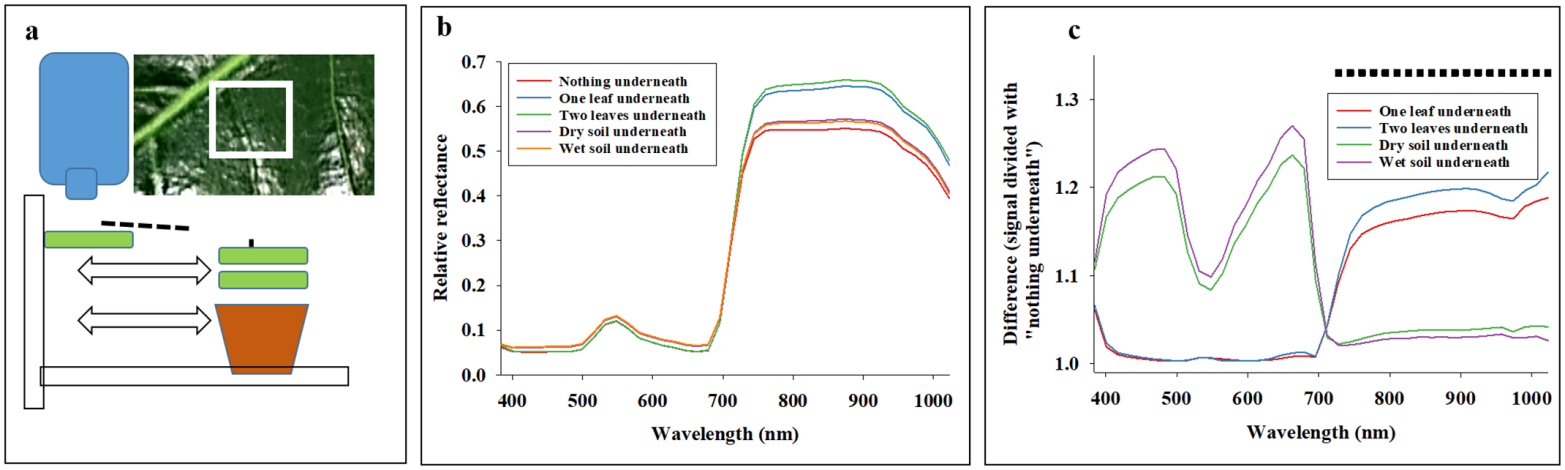
A magnolia leaf was positioned above a benchtop, and hyperspectral imaging data were acquired with nothing underneath, additional Magnolia leaves underneath, or a pot with wet or dry soil underneath (**a**). Average reflectance profiles for all scenarios in 40 spectral bands from 385 to 1024 nm are presented (**b**). Difference in average reflectance values (**C**) represents the difference between nothing underneath compared to other scenarios. Analyses of variance were used to compare differences in all 40 spectral bands, and black squares represent spectral bands in which presence of one or two Magnolia leaves underneath caused a significant increase in average reflectance (P < 0.05).

As a second study of penetration, reflectance profiles were acquired from a magnolia leaf (*Magnolia grandiflora*) in a fixed and suspended position, and either additional magnolia leaves (one or two) or a pot with soil (dry or wet) was placed underneath (Fig 2a). A magnolia leaf is thick and waxy on the surface, so it could be considered considerably less sensitive to potential penetration than thinner leaves of most economically important crops (i.e. wheat, canola, tomato, potato, soybean, etc). Average reflectance signals were acquired from the adaxial side of a magnolia leaf in a fixed position with: 1) nothing underneath, 2) a second and/or a third magnolia leaf immediately underneath, and 3) placement of a pot with wet or dry soil underneath. Analyses of variance (Proc glm) of 12 replications were performed, in which average reflectance from a Magnolia leaf without any objects underneath was compared with average reflectance after placing objects underneath. With four scenarios (One or two additional magnolia leaves immediately underneath or a pot with wet or dry soil underneath) and 40 spectral bands, a total of 160 analyses of variance were performed.

### Imaging of adult mosquitoes

**Fig 3:**
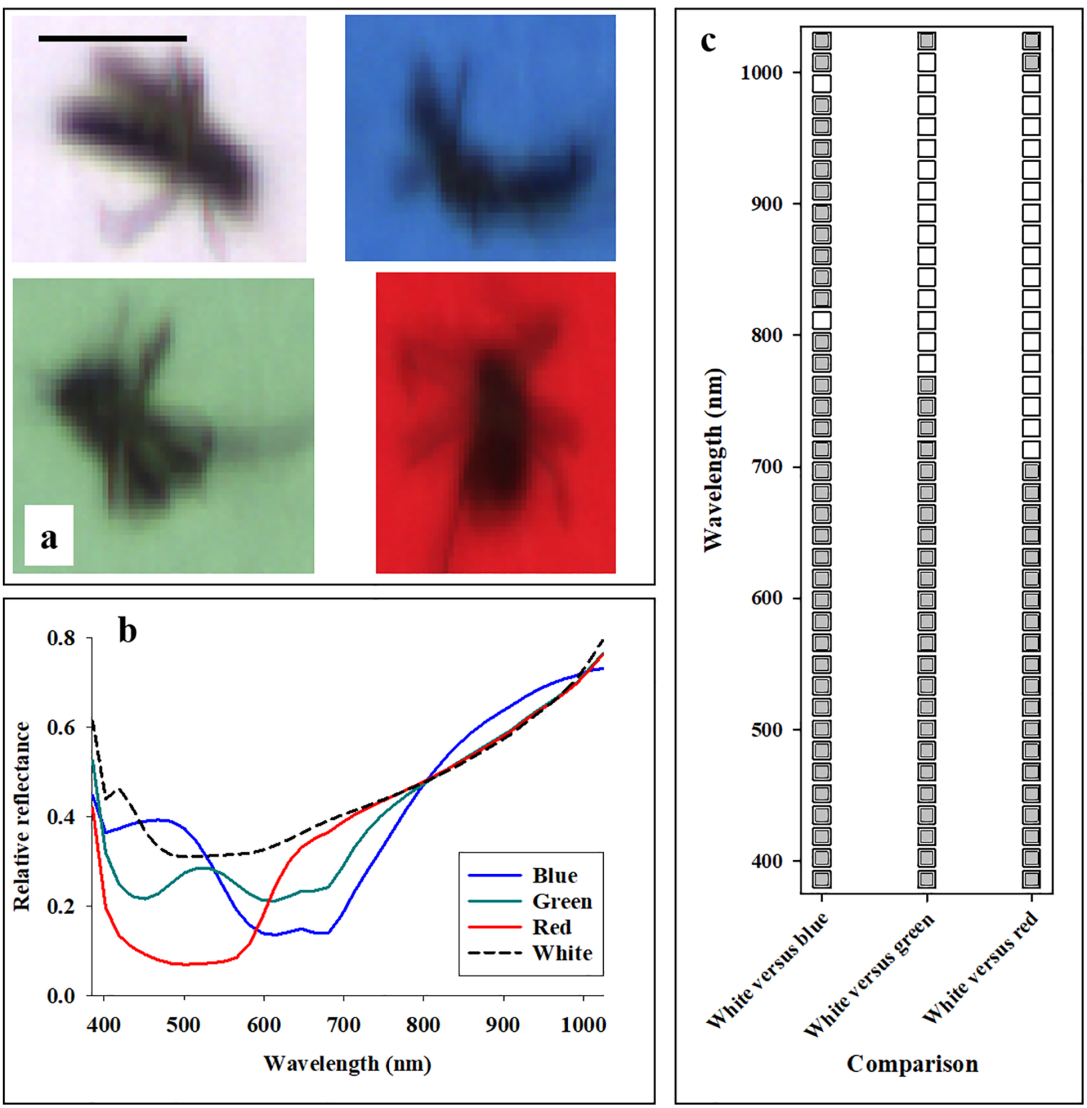
Hyperspectral imaging data were acquired from adult mosquitos (*Aedes albopictus*) placed on top of different colored background paper (**a**). Bar in top left equals scale of 5 mm. After excluding paper background, average reflectance profiles from mosquito bodies were presented (**b**). Average reflectance values in 40 spectral bands from 385 to 1024 nm from adult mosquitoes placed on white paper were compared statistically with reflectance values acquired from adult mosquitoes placed on top of blue, green, or red paper (**c**). Grey color represents significant difference at the 0.05-level.

As a final study of penetration, 20 adult mosquitoes (*Aedes albopictus*) were placed on top of paper in four different colors: white, blue, green, and red (Fig 3a). That is, the exact same 20 adult mosquitoes were imaged four times (placed on different sheets of paper. After hyperspectral imaging, radiometric filtering procedures described in previously published studies [20, 22–25] were deployed, so that background (paper) was excluded. Analyses of variance (Proc glm) of 20 replications were performed, in which average reflectance from adult mosquitoes with white paper as background was compared with average reflectance when colored paper was used as background. With three colors of background paper (blue, green, and red) and 40 spectral bands, a total of 120 analyses of variance were performed.

### Imaging of Skittles

**Fig 4:**
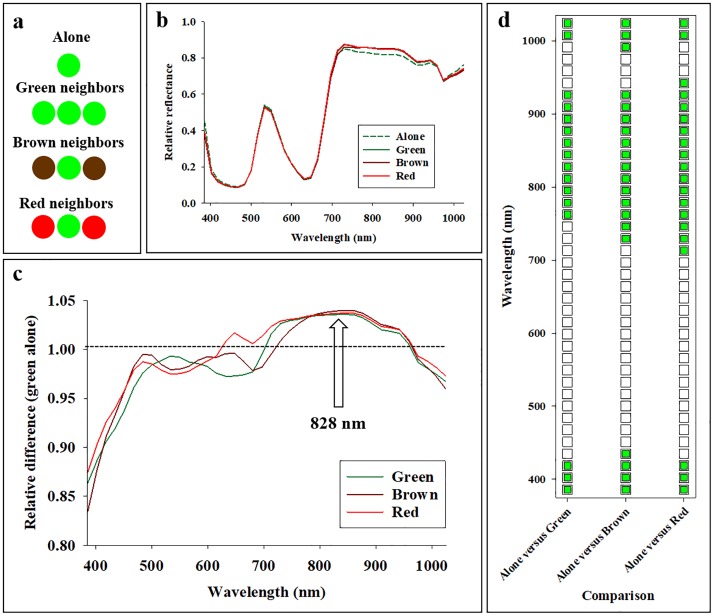
Hyperspectral imaging data were acquired from green pieces of candy (Skittles) under four different scenarios (**a**): 1) no other Skittles (Alone), 2) two neighboring green Skittles (green neighbors), 3) two neighboring brown Skittles (brown neighbors), and 4) two neighboring red Skittles (red neighbors). Average reflectance profiles in 40 spectral bands from 385 to 1024 nm are presented (**b**). Average reflectance (**c**) and statistical analyses (**d**) of difference (average reflectance profiles from scenarios with neighbors divided by average reflectance profiles without neighbors) in 40 spectral bands from 385 to 1024 nm. Grey color represents significant difference at the 0.05-level.

In a study of scattering, average reflectance signals were acquired from green pieces of candy (Skittles, the Wrigley Company) under four different scenarios (Fig 4a): 1) a single green Skittle (denoted “Alone”), 2) Two green Skittles neighboring the green single Skittle (denoted “Green neighbors”), 3) Two brown Skittles neighboring the green single Skittle (denoted “Brown neighbors”), and 4) Two red Skittles neighboring the green single Skittle (denoted “Red neighbors”). The purpose of this experiment was to examine to what extent the presence of neighboring Skittles of different colors affected the average reflectance profile of the green Skittle. Without scattering, the average reflectance profile acquired from the green Skittle in the middle should not be affected by presence and color of neighboring objects. Analyses of variance (Proc glm) of 10 replications were performed, in which average reflectance from the green Skittle alone was compared with average reflectance from the target Skittle when neighboring Skittles were present. With three neighboring scenarios (green, brown, and red) and 40 spectral bands, a total of 120 analyses of variance were performed.

### Imaging of mosquito eggs

**Fig 5:**
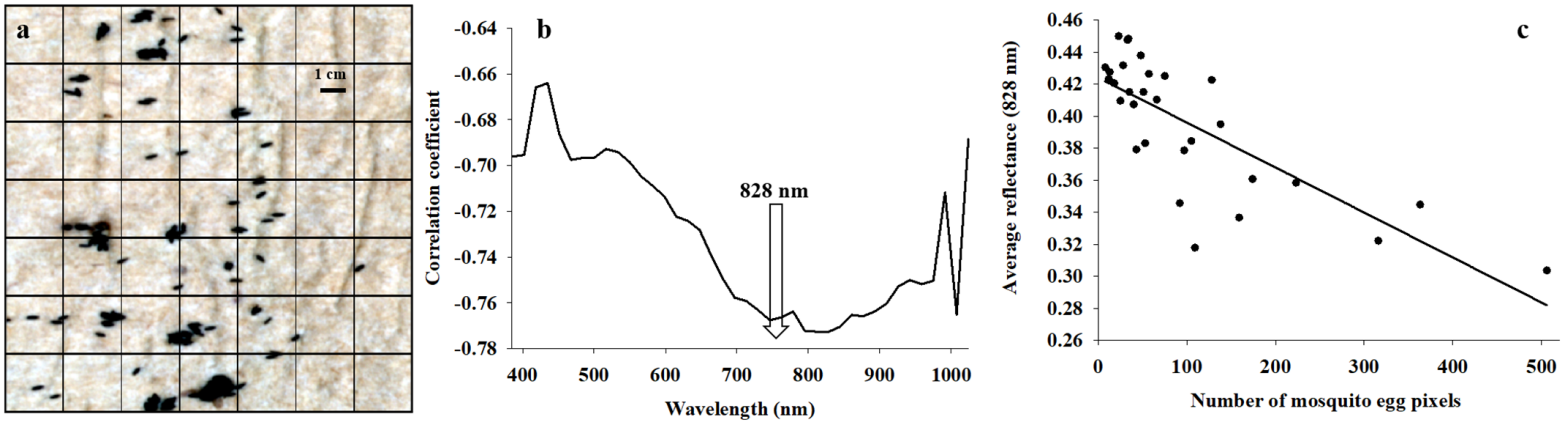
Hyperspectral imaging data were acquired from a piece of paper with mosquito eggs (*Aedes albopictus*) oviposited in random clusters (**a**). The image was divided into 49 squares, and for each square the number of pixels representing mosquito eggs was determined. In addition, the average reflectance was calculated for pixels representing mosquito eggs. Correlation analysis was used for each of the 40 spectral bands from 385 to 1024 nm, and all spectral bands showed a negative correlation between number of pixels representing mosquito eggs and average reflectance (b). Strongest negative correlation was observed at 828 nm (**c**).

In a second study of scattering, reflectance profiles were acquired from mosquito eggs (*Aedes albopictus*), which had been oviposited onto a piece of brown paper (Fig 5a). The entire hyperspectral image cube of mosquito eggs was equal to 122,500 pixels, which were divided into 49 squares. Importantly, the female mosquitoes had oviposited the eggs with varying degree of aggregation, so that both individual eggs and also small and larger clusters of eggs were present. All eggs are oviposited by females in a single layer, so there was a linear correlation between number of eggs and number of pixels representing mosquito eggs. Thus, this data set was considered very suitable for determining the relative effect of egg cluster size on average reflectance profiles. Without the presence of scattering, average reflectance profiles from mosquito eggs should be the same, irrespectively of egg cluster size.

The hyperspectral image of mosquito eggs represented a square of 122,500 pixels (350 by 350), and the total image was divided into 49 equally sized squares (2500 pixels each). A radiometric filter was applied, so that brown background was excluded. Afterwards, average reflectance of pixels representing mosquito eggs in the 40 spectral bands was determined for each square. Only 30 of the 49 squares contained mosquito egg pixels, and for each spectral band a correlation analysis (proc corr) of the relationship between average reflectance and number of pixels representing mosquito eggs was conducted. Under the assumption of no scattering, average reflectance should not be affected by the number of pixels representing mosquito eggs. However under the assumption of scattering significantly affecting the reflectance signals, one would assume, in this case with dark objects (mosquito eggs), a negative correlation between reflectance signals and the numbers of pixels representing mosquito eggs.

## Results

### Imaging of colored dots covered with sheets of white paper

Average reflectance profiles from the three colored dots (no sheets of white paper placed on top) are presented in Fig 1c. For comparison, the average reflectance profile from white paper is also included. It is seen that: 1) average reflectance from blue dots is consistently below that of white paper across the examined spectrum, 2) average reflectance from green dots is similar to that of white paper in spectral bands from 800–1024 nm, and 3) average reflectance from red dots is similar to that of white paper in spectral bands from 650–1024 nm. Fig 1d shows the estimated range (coefficient *c* in Eq 1) in all 40 spectral bands, and several noteworthy trends are highlighted: 1) In spectral bands from 385–600 nm and for all three colored dots, the range was 1–2 sheets of white paper which suggested that penetration in these spectral bands was less than 0.3 mm. 2) Regarding reflectance data acquired in spectral bands from about 850–1024 nm from blue dots, the range was 5–7 sheets of white paper. Thus, the results suggest that penetration in these spectral bands was less than 0.8 mm. 3) Regarding reflectance data acquired in spectral bands from about 850–1024 nm from green dots, the range was 15–18 sheets of white paper. Thus, the results suggest that penetration in these spectral bands was less than 2.0 mm. 4) Regarding reflectance data acquired in spectral bands from about 650–1024 nm from red dots, the range was 17–31 sheets of white paper. Thus, the results suggest that penetration in these spectral bands was less than 3.2 mm.

The results presented here suggest that the choice of background color underneath target objects can greatly impact both quality and robustness of proximal remote sensing data. Moreover, to avoid possible penetration interference by colors underneath, a blue background should be preferred over a red background. In addition, it was shown that effects of colored dots were very similar in spectral bands from 385–600 nm, and that penetration in these spectral bands was negligible (less than 0.3 mm). However, it was also shown that acquired reflectance signals penetrate as far as 3.2 mm (31 paper sheets) in spectral bands from 800–900 nm, when the colored dot underneath is red. In the same spectral range (800–900 nm), the penetration only appeared to be about 2 mm when the colored dot underneath is green and about 0.8 mm when the colored dot underneath is blue.

### Imaging of magnolia leaves

Average reflectance profiles show that placing either pots with soil or additional leaves caused an increase in reflectance across the examined spectrum (Fig 2b). Fig 2c shows the relative change, and it is seen that the strongest response (increase in reflectance) to soil in pots was detected in spectral bands between 400–500 nm and between 600–700 nm. In comparison, the strongest response to additional magnolia leaves placed underneath was observed in spectral bands from 720–1024 nm. Analyses of variance were conducted for all 40 spectral bands, and it was found that: 1) Although there was a considerable increase in average reflectance in spectral bands between 400–500 nm and between 600–700 nm, there was no significant effect of placing a pot with neither dry nor wet soil underneath the imaged magnolia leaf. 2) Spectral bands from 728–1024 nm showed a highly significant (P < 0.001) increase in average reflectance (Fig 2c), when placing either one or two magnolia leaves immediately underneath the imaged magnolia leaf.

### Imaging of adult mosquitoes

Average reflectance profiles acquired from adult mosquitoes showed distinct responses to the color of the paper underneath (Fig 3b): 1) adult mosquitoes placed on top of blue paper had high reflectance in spectral bands from 400–500 nm (blue light), 2) adult mosquitoes placed on top of green paper showed a distinct reflectance peak in spectral bands near 540 nm (green light), and 3) adult mosquitoes placed on top of red paper had high reflectance in spectral bands from 600–700 nm (red light). It is seen that all average reflectance profiles have very similar reflectance values at 800 nm. Fig 3d shows the statistical results from the pairwise analyses of average reflectance acquired from adult mosquitoes placed on white paper compared with each of the three additional colors of background paper, and it is seen that: 1) blue color background paper caused a significant different in average adult mosquito reflectance in 38 of the 40 spectral bands, 2) green color background paper caused a significant different in average adult mosquito reflectance in spectral bands from 383–780 nm, and 3) red color background paper caused a significant different in average adult mosquito reflectance in spectral bands from 383–700 nm.

### Imaging of Skittles

Average reflectance profiles were acquired from green target Skittles showed that presence of neighboring Skittles caused a slight but detectable increase in reflectance in spectral bands from 700–950 nm (Fig 4b). Fig 4c shows the relative difference (average reflectance profiles from scenarios with neighboring Skittles were divided with that from the target Skittle being alone), and several trends are highlighted: 1) presence of neighboring Skittles, irrespectively of color, caused a marked decrease in relative reflectance in spectral bands from 385–480 nm, 2) distinct color-specific reflectance responses were observed in spectral bands from 600–700 nm, and 3) presence of neighboring Skittles, irrespectively of color, caused an increase in relative reflectance in spectral bands from 750–850 nm. Regarding the latter trend, it is seen that the increase in relative reflectance in spectral bands near 828 nm was about 4%. Fig 4d shows the statistical results from the pairwise analyses of average reflectance acquired from green Skittles alone and with presence of neighboring Skittles, and it is seen that: 1) irrespectively of color, there was a significant decrease in relative reflectance in response to presence of neighboring Skittles in the spectral bands near 400 nm, 2) presence of neighboring green Skittles cause a significant increase in relative reflectance in spectral bands from 770–930 nm and a significant decrease in relative reflectance in spectral bands from 1000–1024 nm, and 3) presence of either brown or red neighboring Skittles cause similar significant changes in relative reflectance as seen with green neighbors, but the spectral range of effect was slightly wider, especially with red neighboring Skittles.

To summarize the results from this experiment, it was demonstrated that presence of neighboring Skittles significantly reduced and increased average reflectance in different portions of the examined spectrum. In general, the color of neighboring Skittles appeared to be less important, as the three scenarios with neighboring Skittles caused similar relative reflectance responses. However, presence of red neighboring Skittles caused a significant increase in relative reflectance in spectral bands from 700–940 nm, while presence of green neighboring Skittles caused a significant increase in relative reflectance in spectral bands from 770–930 nm. Thus, important color-specific responses of neighboring Skittles were indeed detected.

### Imaging of mosquito eggs

Fig 5a shows the individual and clusters of mosquito eggs, and of the 122,500 pixels, only 3,072 pixels represented mosquito eggs (2.5%). Of the 49 squares, 30 contained mosquito eggs, and the number of pixels per square representing mosquito eggs varied from 8–506 (average = 102.4). The correlation coefficients of the relationship between average reflectance and number of pixels representing mosquito eggs were highly negative in all 40 spectral bands, especially at 828 nm (Fig 5b and 5c) (df = 1,29, adjusted r^2^-value = 0.583, F-value = 41.496, P-value < 0.001). That is, the radiometric signals acquired from mosquito eggs were darker when more mosquito eggs were present within a square (a higher number of pixels representing mosquito eggs). Thus, this simple image analysis provided clear evidence of scattering influencing the relative signal acquired across the entire spectrum, and particularly in the near infrared region. Furthermore, the strong reflectance response in spectral bands from 770–880 nm is consistent with the reflectance response observed in the experiment with Skittles.

## Discussion

Penetration and scattering are well-known optical phenomena by optical physicists and others involved in the studies of the theories behind spectroscopy. In addition, they are well-known and actually used in the medical field as part of developing disease diagnosis and image-guided surgery [11]. However, these optical phenomena deserve more attention by the growing spectrum of researchers outside the biomedical field applying proximal remote sensing technologies in engineering and biological sciences. In this study, it was demonstrated that both optical phenomena significantly affect average reflectance values in spectral bands from 385 to 1024 nm. In the experiment with white paper sheets on top of colored dots, it was demonstrated that average reflectance signals penetrate through a minimum of five sheets of normal paper spectral bands from 800 to 950 nm, especially if the colored dot is red. In the experiment with suspended magnolia leaves, spectral bands from 700 to 1024 nm had significantly higher average reflectance when additional leaves placed underneath. In the experiment with adult mosquitoes placed on top of colored paper, it was seen that spectral bands from 383–700 nm were particularly sensitive to background color. Regarding scattering, the experiment with Skittles showed reflectance values in spectral bands near 400 nm and from 770–930 nm were particularly sensitive to presence of neighboring Skittles. Finally, the analysis of an image of mosquito eggs showed that density of eggs affected the average reflectance across the examined spectrum, especially in spectral bands from 770–930 nm.

There are numerous studies describing the underlying optical physics of both radiometric penetration [11, 26] and scattering [11, 15, 16, 27] with respect to their effect on proximal remote sensing data. Lu and Fei (12) provide a comprehensive review of both the optical phenomena and applications of proximal remote sensing in the biomedical field. The results presented in this study corroborate existing knowledge about penetration of reflectance signals in the near infrared spectrum (highest in spectral bands from 700 to 1024 nm) due to tissue’s low absorption [28]. In some medical applications of imaging technologies, changes in radiometric penetration at a specific wavelength is the response variable of interest. For instance, it has been demonstrated that the radiometric penetration at 630 nm is 3.42 mm for healthy lung tissue but 2.81 mm for lung tissue with a tumor [26]. In addition, it has been shown that the penetration of radiometric energy into fruits and vegetables is several millimeters, and that it varies with wavelengths [29]. As an example, the penetration in a range of fruits was about 4 mm in spectral bands from 700 to 900 nm, and it was 2–3 mm in spectral bands from 900 to 1,900 nm [28]. In addition, it has been shown that penetration into fruit reached 7.1 mm at 535 nm in plums (*Prunus sp*.) and 13.8 mm at 720 nm for zucchini (*Cucurbita pepo* var. cylindrical) [30]. Challenges associated with penetration should not only be of concern to users of proximal remote sensing technologies; this phenomenon also affects airborne remote sensing applications. In airborne remote sensing applications, it is well known that both soil background and layers of leaves within a canopy affect acquired reflectance data [31]. Moreover, crop leaves are only a few millimeters thick, and it is virtually impossible to control for superimposed leaves and/or effects of soil features underneath the crop canopy. Consequently, penetration may have profound impact on the spectral repeatability of both airborne and proximal applications of remote sensing technologies.

Somewhat surprisingly, this study based on proximal remote sensing data appears to be the first in which experimental data were used to investigate the relative effect of scattering across a broad radiometric spectrum. However, scattering is known to have considerable impact on airborne remote sensing signals. As an example, it has been estimated that, due to scattering, less than half of the signal recorded by the Landsat’s multispectral scanner system originates from the imaged pixel itself, while the remainder of the reflectance signal is derived from surrounding pixels [32]. The results presented here suggest that scattering can greatly influence the robustness of reflectance signals, and that classification accuracies may vary considerably depending on the density of objects being classified. It should be mentioned that Skittles have a rounded and somewhat shiny surface, which likely contributes to the significant effect of neighboring Skittles on the average reflectance signals acquired from the green Skittle in the middle. However, many objects have similarly shiny surfaces (insects, seeds, crystals, etc), so the Skittle experiment is considered relevant to a broad range of engineering and biological applications.

Despite a rapidly growing interest in use of proximal remote sensing technologies across a wide spectrum of engineering and biological disciplines, there are surprisingly few studies focusing on how effects of penetration and scattering can be minimized and how to correct for them when imaging data is being acquired. Furthermore, these optical phenomena are hardly ever mentioned as justifications for particular experimental designs, nor are they mentioned as possible contributors to low or inconsistent classification results. Overall, this series of experiments showed that spectral bands in the near infrared region are particularly sensitive to both penetration and scattering. Finally, the experiment with adult mosquitoes placed on top of different colored paper highlighted that spectral bands from 383–700 nm may also be very sensitive to imaging conditions. Due to penetration, the thickness of target objects should be taken into consideration, and it is important to standardize positioning of objects so that variability in background surface underneath is not causing noise in the reflectance signals. It is argued that characterization of the relative sensitivity of reflectance data to penetration and scattering is an important step towards increasing the radiometric robustness of signals and therefore the reliability of proximal remote sensing technologies as tools used to classify engineering and biological objects.
